# FFA Patient Profile Analysis Based on the Authors’ Observations and a Review of the Literature—An Original Survey

**DOI:** 10.3390/jcm14124346

**Published:** 2025-06-18

**Authors:** Michał Owczarek, Magdalena Jałowska, Agnieszka Mariowska, Wiktoria Grochowska, Joanna Szyszkowska, Daria Metelkina, Maciej Marek Spałek

**Affiliations:** 1 Department of Dermatology, Poznan University of Medical Sciences, 60-355 Poznan, Poland; mjalowska@ump.edu.pl (M.J.); agnieszkamariowska18@gmail.com (A.M.); wiktoriagr6467@gmail.com (W.G.); dariametelkina21@gmail.com (D.M.); mspalek@ump.edu.pl (M.M.S.); 2 Department of Psychology, University of Warsaw, 00-183 Warsaw, Poland; j.szyszkowska3@student.uw.edu.pl

**Keywords:** frontal fibrosing alopecia, scarring alopecia, clinical profile, survey study, menopause, reproductive system malignancy, hypothyroidism, hair dye, scalp itching, sunscreen

## Abstract

**Background/Objectives**: Frontal fibrosing alopecia (FFA) is a scarring alopecia with an unclear aetiology, primarily affecting postmenopausal women. This study aims to identify potential risk factors contributing to FFA development and progression, as well as provide a clinical profile to aid in the differential diagnosis. **Methods**: The study included 19 women diagnosed with FFA. The participants completed a 20-question survey based on a literature review of potential risk factors. Statistical analyses were performed to investigate the associations between patient characteristics and FFA. **Results**: All patients were female and their mean age was 60.58 years (SD = 12.81). In 63.1% of the cases, FFA onset occurred postmenopause, with a mean latency of 8.17 years. In the majority of cases, the diagnostic delay exceeded five years. The average menarche age was 13.68 years (SD = 2.06), whereas late menarche (≥15 years) was found in two subjects. A history of reproductive organ or breast malignancy was reported by 42.1% of the patients, which frequently required surgery. Most subjects did not receive hormone replacement therapy, or hormonal contraception. The most prevalent comorbidity was hypothyroidism (89.47%). Although smoking was rare among the subjects, hair colouring was quite common, yet no participant underwent scalp aesthetic procedures. In 47.4% of cases, scalp itching or pain was present. Sunscreens were frequently used, mostly on a daily or seasonal basis. **Conclusions**: FFA predominantly affects women in their early 60s, often following the menopause. In our study, a tendency toward an early menopause and an above-average menarche age of the subjects was observed. In the analysed group, only reproductive and breast cancers were reported, which requires further investigation. Frequent β-blocker use, second only to levothyroxine, may suggest that they play a role in FFA pathogenesis. Itching and pain of the scalp may contribute to the correct diagnosis, although these symptoms are not universal. Moreover, sunscreens were indicated as a potential trigger, yet avoiding them should not be routinely recommended due to the risk of carcinogenesis. The variability in the diagnostic delay emphasises the need for increasing clinician awareness and conducting further research.

## 1. Introduction

Frontal fibrosing alopecia (FFA) represents a rare and relatively recently recognised disease, first described in 1994 by Steven Kossard [1,2]. It is a progressive form of scarring hair loss, characterised by hair loss predominantly in the frontal and temporal scalp regions [3]. Although it primarily affects postmenopausal women, FFA was also reported in men and premenopausal women [4,5]. The condition presents with specific symptoms of frontal hairline recession, scalp inflammation, scaling and perifollicular erythema, which contribute to its distinctive clinical presentation [6].

Despite its increasing prevalence, the exact cause of FFA remains unclear and involves a complex interplay of genetic predispositions, hormonal influences, and environmental factors [7]. There are a number of controversial and frequently conflicting hypotheses with regard to the disease aetiology [8]. Currently, the literature highlights significant gaps in the understanding of FFA pathogenesis, where certain studies suggest potential links to autoimmunity and androgen-related mechanisms, whereas other emphasise environmental triggering factors, such as the use of sunscreens and particular cosmetic products [9,10,11,12,13,14]. FFA is now considered a patterned clinical variant of lichen planopilaris (LPP), an autoimmune disease mediated by folliculotropic cytotoxic T-cells targeting the hair follicle epithelium. Additionally, the diagnostic process may be challenging due to the clinical and histopathological similarities with other scarring alopecias, and therefore requires a thorough clinical evaluation, trichoscopy, and—in some cases—a scalp biopsy [15,16]. Contrary to overt clinical scarring, the fibrotic changes seen in FFA often manifest as subtle, pale, featureless skin, reflective of histological fibrosis rather than gross dermal remodelling. These conflicting viewpoints indicate the need for further research to elucidate these associations in order to fully determine its aetiology and exact nature, and thus improve the diagnostic as well as therapeutic processes available for FFA patients.

The objective of this study is to describe the profile of a typical FFA patient and thereby assist clinicians with the diagnostic process, which in most cases remains complex. Moreover, by using patient-reported data, it also attempts to explore potential links between FFA and various environmental factors in order to clarify key correlations and gain insights into the pathogenesis of FFA.

By means of integrating the clinical and patient perspectives, our research seeks to enhance the understanding of this condition and ultimately improve clinical outcomes for the affected individuals. This paper also provides an overview of the current knowledge regarding FFA, addresses the diagnostic challenges, as well as offers relevant insights for both practitioners and researchers.

## 2. Materials and Methods

The survey was conducted at the Department of Dermatology at the Heliodor Swiecicki Hospital (Poznan University of Medical Sciences Clinical Hospital) between May 2023 and May 2024, and involved patients suffering from frontal fibrosing alopecia who were referred for a dermatology consultation.

The eligibility criterion was the diagnosis of frontal fibrosing alopecia and treatment at the Department of Dermatology at Poznan University of Medical Sciences. All subjects agreed to participate in the study and provided a written consent. The research was approved by the Bioethics Committee of Poznan University of Medical Sciences (approval number 304/23, 6 April 2023). 

The diagnosis of FFA in all included patients was established by experienced dermatologist based on a combination of clinical, trichoscopic and histopathological criteria, in accordance with current international diagnostic guidelines. Trichoscopy was performed in all cases and in every case revealed findings characteristic of FFA. In selected cases, a confirmatory scalp biopsy was conducted demonstrating histopathological hallmarks of FFA and supporting the clinical and trichoscopic diagnosis.

The participants were asked to fill out the original questionnaire, which contained 20 questions created by the authors on the basis of the latest literature review, regarding the factors which affected the development of FFA. A literature search was based on the PubMed and Google Scholar databases, and included studies published between 2005 and 2025.

The format of the questionnaire comprised yes/no and open-ended questions, which pertained to menstruation, duration of the condition, oncological diseases, administered medications, treatments used—i.e., aesthetics medicine procedures on the scalp or hair colouring—as well as the patients’ smoking habits and sunscreen use. Ultimately, 19 subjects were included in the study.

The patient questionnaire used in this study is provided in Supplementary Materials File S1.

The statistical analysis was performed using SPSS version 28. Due to the small number of participants, only the frequency analysis was applied.

## 3. Results

Our study comprised 19 patients, aged 31 to 75 years (M = 60.58; SD = 12.81) and diagnosed with FFA. The largest group, which included 9 subjects in their 60s (between 61 and 68 years of age), accounted for 47.3% of the analysed group. All participants were female.

At the time of the survey, 15 patients (78.9%) stated that they had already had their last menstrual period, whereas in only 4 (21.1%) subjects the menopause had not started yet. One of the participants, a 33-year-old woman, had undergone a hysterectomy with the removal of one ovary and therefore, for statistical purposes, she was included in the postmenopausal group. Once the aforementioned patient was excluded, the age of the subjects during their last menstruation ranged between 47 and 58 years (M = 51.47; SD = 6.21). Most women experienced the menopause at the age of 50 (*N* = 4; 21.1%), and the largest number of patients within this group entered the menopause between 50 and 55 years o (*N* = 9; 47.4%).

In the case of 12 patients, constituting 63.1% of the analysed group, the first symptoms of alopecia appeared after the menopause. Another 7 individuals (36.9%) had been premenopausal when first symptoms occurred; however, at the time when the study was conducted, 3 of them (15.8%) were already postmenopausal, while 4 patients (21.1%) experienced menstrual cycles both at the time of the first symptoms and throughout the study.

As for the symptoms that developed in the patients following the menopause, they appeared between six months and even up to 20 years following the last period. The mean interval between the menopause and the first symptoms of alopecia was 8.17 years (SD = 6.68).

Notably, only one patient (5.3%) was correctly diagnosed with FFA at her first medical appointment. The highest percentage of patients (*N* = 4; 21.1%) received an appropriate diagnosis one year after the onset of alopecia symptoms; however, in a significant proportion of the patients, the time until the proper diagnosis was considerably longer. The diagnosis of FFA was established after 5 years or more in 8 patients (*N* = 8; 42.2%) (M = 5.45; SD = 7.17), and the longest period from the onset of the symptoms to the diagnosis amounted to 27 years.

The patients in our study entered menarche at ages ranging from 9 to 17 years (M = 13.68; SD = 2.06). The largest group (*N* = 7; 36.8%) experienced the first menstruation at the age of 14; the next most numerous group (*N* = 4; 21.2%) started menstruating at the age of 15. Moreover, 2 participants (10.5%) in the study group reported menarche over the age of 15—both were 17, which could be considered a late menarche. The majority of the analysed individuals (*N* = 13; 68.4%) indicated the first menstruation above the age of 13. Only one patient (5.3%) started menstruation at the age of 9, which could be classified as early menarche. Interestingly, the majority of our patients menstruated regularly (*N* = 15; 78.9%), whereas only 4 subjects (21.1%) showed irregular menstruation.

The majority of the surveyed women could not provide the age at which their closest female relatives entered menopause (*N* = 10; 52.6%). In one case, the youngest family member experienced the menopause at the age of 42, whereas the oldest experienced it at the age of 55. The mean age for menopause in the family of the analysed female patients was 49 years of age (SD = 4.44).

In the studied group, 15 (*N* = 15; 78.9%) participants were pregnant at least once, while 4 (*N* = 4; 21.1%) subjects had never conceived. The number of suies ranged from one to five, and the majority of the respondents were pregnant twice (*N* = 10; 52.6%). Only one pregnancy resulted in a miscarriage, and it involved a patient who had been pregnant twice.

The majority of the studied group had no history of cancer, although 8 (*N* = 8; 42.1%) patients listed various types of cancer or precancerous conditions in their medical history. The reported types of cancer and their frequency are presented in Table 1.

More than half of the participants in our study (*N* = 11; 58.2%) had undergone at least one surgical intervention, which predominantly comprised gynaecological procedures. Several patients had been subject to more than one type of procedure. The surgical procedures and their frequency are summarised in Table 2.

In the case of one patient diagnosed with breast cancer, the treatment included mastectomy, as well as radio- and chemotherapy with tamoxifen.

Most patients had not received hormone replacement therapy (HRT) (*N* = 15; 78.9%); however, 4 subjects (*N* = 4; 21.1%) reported using HRT in the form of oestriol tablets (*N* = 1; 5.3%), oestradiol transdermal system (*N* = 1; 5.3%) and soya bean extract tablets (*N* = 1; 5.3%). One patient did not specify the medication she took. The duration of HRT administration among the participants varied from 6 months to over 20 years.

The majority of the studied group did not take hormonal contraception *(N* = 12; 63.2%). Of the 7 patients (*N* = 7; 36.8%) who did, 5 used birth control pills (*N* = 5; 26.3%), 2 used intrauterine devices (*N* = 2; 10.6%), and 1 patient (5.3%) was unable to answer the question regarding the form of hormonal contraception she had received.

Furthermore, 17 subjects (*N* = 17; 89.47%) suffered from other diseases apart from FFA. According to the analysed data, the majority of the surveyed women suffered from hypothyroidism. The frequency of the most common comorbidities is presented in Table 3.

Other medical conditions in the studied group included the following: insulin resistance, angina pectoris, tachycardia, peripheral venous insufficiency, gastrointestinal motility disorders, hepatic steatosis, cerebrovascular disorders, vertigo, as well as osteoarthritis, allergies to dust and mites, and cataracts.

The patients were also requested to provide information as to which medication they were currently using. Only 3 individuals (*N* = 3; 15.8%) were not taking any drugs, whereas 1 patient did not specify which hypertension medication she received. Levothyroxine was the most commonly administered medication and it was prescribed as a treatment for hypothyroidism in 10 patients (*N* = 10; 52.6%). In turn, 6 patients (*N* = 6; 31.6%) reported taking statins, including 4 who received rosuvastatin (*N* = 4; 21.1%) and 2 who used simvastatin (*N* = 2; 10.5%). One patient used a dietary supplement containing red yeast rice, policosanol, folic acid, coenzyme Q10 and astaxanthin. Several participants received medication for hypertension, where β-blockers represented the predominant drug class (*N* = 4; 21.1%) and included bisoprolol (*N* = 3; 15.8%) and metoprolol (*N* = 1; 5.3%). In total, 5 patients (*N* = 5; 26.3%) reported taking diuretics (in the form of a single or combination drug), including 3 (*N* = 3; 15.8%) subjects using indapamide, which is classified as belonging to the sulphonamide group, and 2 (*N* = 2; 10.5%) participants who received hydrochlorothiazide in combination with losartan, which belong to the thiazide and sartan groups, respectively. Another 3 (*N* = 3; 15.8%) patients received calcium antagonist medications, among whom 2 (*N* = 2; 10.5%) individuals were administered lercanidipine and 1 (*N* = 1; 5.3%) patient used lacidipine. One participant (*N* = 1; 5.3%) received ramipril, which is one of the angiotensin-converting enzyme inhibitors. Other less frequently administered drugs included metformin, ivabradine, calcium dobesilate, trimebutine, pantoprazole, timonacicum, vinpocetine, flunarizine and betahistine.

None of the studied patients reported undergoing any medical or aesthetic procedures on the scalp, such as hair transplants, prior to the onset of scarring alopecia symptoms. Furthermore, the majority of the subjects (*N* = 14; 73.7%) admitted to dying their hair, as compared to 5 (*N* = 5; 26.3%) individuals who denied using hair colouring products. As for unusual or aggressive hair treatments, such as hair extensions or frequent use of high-temperature styling devices (e.g., straighteners, curlers), they were reported by 5 (*N* = 5; 26.3%) respondents. Specific treatments included keratin treatments (*N* = 1; 5.3%), frequent or occasional straightening (*N* = 2; 10.6%), as well as regular styling with high-temperature appliances, such as thermal rollers (*N* = 2; 10.6%).

In terms of tobacco smoking, the vast majority of the participants indicated that they had never smoked in the past (*N* = 15; 78.9%). In turn, 3 (*N* = 3; 15.8%) patients were current smokers and 1 (*N* = 1; 5.3%) patient was a former smoker.

Itching or scalp pain was noted by 9 (*N* = 9; 47.4%) patients, whereas 10 (*N* = 10; 52.6%) subjects did not experience these symptoms.

Sunscreen use was reported by the vast majority of the respondents (*N* = 15; 78.9%), with SPF values fluctuating between 10 and 100. Among the patients who used sunscreens, 6 (*N* = 6; 31.6%) women applied SPF 50, 5 (*N* = 5; 26.3%) participants opted for SPF 30, 1 (*N* = 1; 5.3%) subject reported using SPF 20, 1 (*N* = 1; 5.3%) patient—SPF 10, and 1 (*N* = 1; 5.3%) respondent—SPF 100. Additionally, there was also 1 patient who did not remember which SPF value she used. The frequency of using sunscreen was as follows: 7 (*N* = 7; 36.8%) patients used sunscreen daily, 8 (*N* = 8; 42.1%) respondents used sunscreen seasonally during the summer, and only 4 (*N* = 4; 21.1%) participants did not use sunscreen at all.

## 4. Discussion

Our findings confirm that scarring alopecia primarily affects postmenopausal women in their early 60s, consistent with previous studies [17,18]. It is rarely observed in men [19] and is considered a condition primarily found in postmenopausal women [20] at a mean age of 62–64 years [21], which aligns with our results.

The mean age of menopause in our cohort aligns with that reported for Caucasian women in industrialised countries, typically between 52 and 55 years [22]. None of the patients experienced menopause before age of 45, which is considered early and has been identified as a risk factor for FFA [23].

Our results support previous findings showing that FFA symptoms typically appear after the last menstruation [24], with onset occurring 2 to 12 years postmenopause, and a mean latency of 5.5 to 10 years [25].

Notably, FFA was correctly diagnosed at the first medical visit in only one case, while for many patients the diagnostic process took considerably longer. This variability may reflect differences in clinical presentation as well as the age and experience of the physicians involved. Hence, it seems essential to conduct further research to understand the relationship between these factors.

These observations highlight the need for further research on FFA to improve diagnosis, treatment, and clinician awareness—particularly among dermatologists. This seems even more relevant considering the increasing global prevalence of this rare entity [9,26].

Menarche typically occurs between ages 10 and 16, with a mean of 12–13 years [27]. It is considered early before age 10 and late after age 15 [28]. Our analysis suggests that patients with FFA tend to experience menarche at the upper end of the normal range or later. As no comparable data were found in the literature, our study may be the first to explore this aspect, though larger cohorts are needed to confirm this observation.

Interestingly, most participants reported regular menstrual cycles. While this may seem unexpected—given that irregular cycles may indicate excessive androgenic activity, which contribute to the development of such conditions as female pattern hair loss (FPHL)—androgens may also play a partial role in FFA pathogenesis [29]. Irregular cycles are associated with increased severity of various dermatological disorders, e.g., acne and atopic dermatitis [30]. However, our findings align with studies suggesting that FFA and follicular scarring may result from androgen deficiency, including low levels of androstenedione, DHEA-S, and DHEA, which regulate PPAR-c receptors involved in TGFB1-mediated scarring [31,32,33].

The mean age of menopause onset in close relatives of the patients was not only lower than that of the FFA group but also below the population average [34]. Notably, one relative experienced menopause before age 45, meeting the criteria for early menopause [35,36]. Previous studies indicate that early menopause is a risk factor not only for FFA development but also for earlier symptom onset [14].

According to both our findings and other literature data, individuals with FFA are more likely to experience a late menarche and an early menopause [37]. However, this observation requires further research on a larger group of patients.

We observed that a significant proportion of respondents reported a history of cancer, all of which involved reproductive organs or breast, and had undergone at least one surgical procedure. This association warrants further investigation, particularly as prior oophorectomy or hysterectomy have been identified as potential risk factors for FFA development [38,39]. Most surgeries in our group were gynaecological, including two hysterectomies and one ovarian cystectomy. Moreover, as shown by Moreno-Arrones et al. prior tamoxifen therapy significantly increases the risk of FFA (OR 14.89; 95% CI 2.42–91.68) [40].

In our study, the majority of the participants did not receive HRT. Nevertheless, Moreno-Arrones et al. demonstrated a statistically significant correlation between HRT and FFA, possibly due to hormonal changes involving oestrogen and prolactin levels during treatment (OR = 1.76; 95% CI: 1.11–2.8) [41]. Moreover, Tosti et al. reported that 35.7% of FFA patients had been treated with oestrogen-based HRT at the onset of the disease [29], a finding that is consistent with our analysis, as the participants who received HRT were primarily administered medications containing various forms of oestrogens. These observations support the hypothesis that patients with FFA may be more likely to experience early menopause and consequently, seek hormone replacement therapy.

Our analysis showed that most participants had not used hormonal contraception. However, the impact of oral hormonal contraception on the development of FFA remains disputed. Initially, due to their high androgenic index, they were suspected to contribute to androgenetic alopecia. Nonetheless, more recent data suggest that their oestrogen component may have a protective effect by promoting hair growth [40,42,43]. In our cohort, 2 patients reported using an intrauterine device (IUD), whereas Beundia-Castano et al. identified IUD use as a potential protective factor against FFA (OR = 0.22; 95% CI: 0.06–0.84) [40].

We also observed a high prevalence of hypothyroidism among surveyed patients. Similarly, Moreno-Arrones et al. reported an increased incidence of autoimmune diseases, including hypothyroidism, in individuals with FFA—20.8% compared to 13.1% in controls (OR = 1.73; 95% CI: 1.11–2.69) [41]. In another study by Valesky et al., co-occurrence of FFA and hypothyroidism reached 58%, significantly exceeding the rate observed in the general population (6–10%) [44]. Notably, hypothyroidism frequently co-occurs with other types of alopecia, such as alopecia areata, androgenic alopecia, and telogen effluvium [45,46,47].

Hypertension and hypercholesterolemia are common in this age group, and in our cohort, making it difficult to establish a direct link with FFA. Their co-occurrence may be incidental [48,49], although some studies report a frequent overlap with dyslipidaemia and hypertension in FFA patients [50,51].

Although no drug-induced cases of FFA have been reported, β-blockers have been implicated as potential triggers of LPP [20]. In our study, they were the second most commonly used medication after levothyroxine. As FFA is considered a variant of LPP [52], a possible association with β-blockers cannot be excluded. Further studies on larger cohorts are needed to verify this hypothesis.

Scalp aesthetic procedures are rarely recognised as direct risk factors for developing FFA. Nevertheless, several cases following surgeries such as hair restoration or facelifts have been reported [53], possibly due to IFNγ-induced immune privilege collapse, isomorphic reactions, or the Koebner phenomenon [54]. Although identifying potential triggers within such procedures is important, none of our patients had undergone them.

Some studies suggest hair dye use as a possible environmental factor in FFA onset; however, evidence remains inconclusive [8]. Most of our patients used hair dye, while other studies reported significantly lower hair dye use in FFA patients compared to controls [53]. Nevertheless, these data provide no evidence of a cause-and-effect relationship [55].

Although the role of heat and mechanical hair treatments in FFA remains unclear, some studies suggest they may cause scalp inflammation or follicle damage [56]. In our study, few participants reported using such treatments, suggesting they are unlikely primary causes of FFA.

In view of the fact that smoking is associated with a systemic inflammation, it has been assumed to affect certain dermatological disorders; yet, its role in FFA remains unclear [21,23]. The low prevalence of smokers in our study suggests that it is not a major contributing risk factor.

Our findings show that scalp symptoms, including itching or pain, represent frequent, although not universal, characteristics of FFA. The nearly equal distribution of symptomatic and asymptomatic patients reflects clinical variability. Nonetheless, symptoms like pruritus and pain remain minor diagnostic criteria described in the literature [57,58].

The survey showed frequent sunscreen use among patients. Interestingly, certain products, especially those containing physical filters like zinc and titanium dioxide have been suggested as potential environmental triggers for FFA [34,41,55,59,60,61]. However, this association is controversial, as affected individuals may be more diligent about sun protection [59,62]. Given the lack of conclusive evidence for a casual link, routine avoidance of sunscreen in FFA patients is not recommended, especially in Caucasians with photodamaged skin, where sun protection reduces the risk of serious conditions like actinic damage and subsequent skin cancer [63,64].

This study has several limitations that should be acknowledged. Firstly, the small sample size, due to FFA’s rarity and alopecia-related stigma, limits participant availability, so our findings should be viewed as descriptive and exploratory while provided percentages are meant to offer a general sense of trend rather than support definitive conclusions. Secondly, the single-centre design restricts generalisability and broad epidemiological conclusions. Additionally, reliance on self-reported, retrospective data introduces potential recall bias, although trained medical staff assistance was available during questionnaire completion and, wherever possible, key clinical variables were crossed-checked with available medical records to improve reliability. Diagnoses were based on clinical and trichoscopic criteria by an experienced dermatologist, but confirmatory scalp biopsies were performed only in selected cases, possibly limiting diagnostic certainty and allowing overlap with other cicatrical alopecias. Consequently, further multicentre studies with larger cohorts and routine histopathological confirmations are warranted to comprehensively assess potential risk factors and better elucidate the underlying aetiology of FFA.

## 5. Conclusions

Our study confirms that FFA predominantly affects postmenopausal women in their early 60s, who manifest a clear tendency for an early menopause and a higher-than-average age at menarche. Even though FFA remains a poorly understood condition, our findings corroborate the hypothesis that hormonal changes, primarily involving androgens and oestrogen metabolism, may play a role in the disease development. Furthermore, the high prevalence of hypothyroidism is indicative of a potential autoimmune component, and the exclusive occurrence of reproductive and breast tumours in our cohort warrants more-in-depth investigation. Additionally, frequent use of β-blockers raises the question of whether certain medications could influence the onset or progression of FFA. Among environmental factors, smoking appears insignificant, whereas hair dye use and widespread sunscreen application may represent potential disease triggers. However, our findings do not support routine sunscreen avoidance, given its role in photoprotection and cancer prevention. The clinical presentation of FFA varies, thus, rendering the diagnostic process complex and demanding. Scalp itching/pain is frequent, yet not universal; however, another significant challenge seems to be a delayed diagnosis. This, in turn, emphasises the necessity of raising awareness among practitioners in order to facilitate earlier detection and subsequent interventions, which may potentially mitigate the progression of the disease and improve treatment outcomes.

In conclusion, our study provides valuable insights into the clinical profile and the potential risk factors contributing to the abovementioned goal. Nevertheless, due to the relatively small sample size, further large-scale, controlled, multicentre studies are crucial to confirm these associations, and to identify the underlying pathophysiological mechanisms especially the nature of LPP and its role in eliminating hair follicles. A multidisciplinary approach—incorporating dermatology, endocrinology, autoimmunology, as well as pharmacological and cosmetic research—may be the key to developing more effective diagnostic and therapeutic strategies in the future.

## Figures and Tables

**Table 1:** Frequency and type of cancer in the study group.

Types of Cancer	*N*	%
None	11	57.9
Breast cancer	2	10.6
Uterine myoma	1	5.3
Uterine polyp	1	5.3
Hydatidiform mole	1	5.3
Uterine cancer	1	5.3
Ovarian cyst	1	5.3
Ovarian cancer	1	5.3

**Table 2:** Frequency and type of surgical procedures in the study group.

Types of Cancer	*N*	%
None	8	42.1
Thyroid surgery	3	15.8
Hip endoprosthesoplasty	2	10.6
Mastectomy	2	10.6
Hysterectomy	2	10.6
Uterine polypectomy	1	5.3
Uterine cavity lysation	1	5.3
Endometriosis surgery	1	5.3
Caesarean section	1	5.3
Ovarian cystectomy	1	5.3
Ovariectomy	1	5.3

**Table 3:** The most common comorbidities in the study group.

Types of Cancer	*N*	%
None	2	10.6
Hypothyroidism	10	52.6
Hypertension	8	42.1
Hypercholesterolemy	7	36.8

## Data Availability

The raw data that support the conclusions of this study are available on request from the authors. The data are not publicly available due to privacy, or ethical restrictions.

## Supplementary Materials

The following supporting information can be downloaded at https://www.mdpi.com/article/10.3390/jcm14124346/s1: Supplementary File S1: The questionnaire.
