# Supplementary material for: FFA Patient Profile Analysis Based on the Authors’ Observations and a Review of the Literature—An Original Survey
Source: J Clin Med. 2025 Jun 18;14(12):4346. doi: 10.3390/jcm14124346 (PMC12194613; doi:10.3390/jcm14124346)
Supplement: Supplementary file 1 [file jcm-14-04346-s001.zip › jcm-3670003-supplementary.pdf]

Patient’s full name:

Patient’s age:

1. Are you still menstruating? If not, at what age did your last menstrual period occur (i.e., age at menopause)?
2. How long after your last menstrual period did hair loss begin?
3. How much time passed between the onset of hair loss and the diagnosis?
4. What was your age at menarche (first menstruation)?
5. Did/do you menstruate regularly? What was the average cycle length?
6. What was the age of menopause in your female relatives (e.g. mother, grandmother, sisters)?
7. How many pregnancies have you had? How many deliveries and in which years?
8. Have you ever been diagnosed with any malignancy (especially of the reproductive organs)? If yes, when and what treatment was administrated?
9. Have you ever undergone any surgery, including gynaecological procedures (especially total hysterectomy)? If yes, what type and when?
10. Have you ever been diagnosed with breast cancer? If yes, when and what treatment was used?
11. Have you used hormone replacement therapy (HRT)? If yes, what type, for how long, and in what dosage?
12. Have you used hormonal contraception? If yes, what type, for how long, and in what dosage?
13. Have you been diagnosed or treated for any other medical conditions? If yes, please specify.
14. What medications have you been taking regularly? Please specify duration and dosage if possible.
15. Have you ever undergone any aesthetic procedures on the scalp prior to the onset of hair loss, such as hair transplantation or other interventions?
16. Have you dyed your hair in the past or currently do so?
17. Prior to the onset of hair loss, did you undergo any intensive or non-standard hair treatments (e.g., hair extensions, frequent curling or straightening using heat)?
18. Do you or did you smoke cigarettes? If yes, how many per day and for how long?
19. Do you experience any itching or pain of the scalp?
20. Do you use sunscreens? If yes, how often and with what SPF value?
